# The Attitude of the Medical Profession toward Food Preservatives

**Published:** 1904-06

**Authors:** 


### THE ATTITUDE OF THE MEDICAL PROFESSION TOWARD FOOD PRESERVATIVES.*

There is a very preponderating sentiment as expressed by members of the medical profession respecting food preservatives and other foreign substances added to foods to the effect that they are injurious. In most cases this opinion is not based on actual experimental work, but either on a priori conclusions relative to the action of such bodies on the physiological functions or on the evidence presented by others. There are, however, occasional articles from medical men of fine standing and reputation, combatting the generally expressed views on this subject and asserting that the common preservatives in food products, when used in small quantities, are not prejudicial.

For many years this problem has been presented for my consideration in various forms, official and unofficial, and I am now charged, under existing laws, with officially advising the Secretary of Agriculture and other authorities in respect of these matters. The great responsibility of a position of this kind has caused me to re-examine with much care the general literature upon the subject and to ascertain the extent of preponderating testimony in regard to these injurious effects.

When I examine the experimental evidence, in so far as it extends, I find much that is contradictory. For example, in the case of borax I find that the experiments which were conducted by the chemists and physiologists of the Imperial Board of Health of Berlin lead to the conclusion that borax, even in small quantities, is highly injurious. On the other hand, similar experiments conducted by Professor Liebreich in Berlin, Doctors Rosenheim and Tunicliffe in London and Professor Chittenden in this country lead to opposite results. The same difficulties, though to a less extent, are shown in experimental work connected with other preserva-

*By H. W. Wiley, M.D., Chief of the Bureau of Chemistry, United States Department of Agriculture, Washington, D. C.

tives, and the existence of fewer difficulties is probably due to the fact that the experiments with other preservatives have not been conducted so carefully nor by so many workers. In all cases like that of borax, mentioned above, it is difficult to determine in what direction the preponderant testimony leans, whereas with such substances as salicylic acid and formaldehyde the determination is less difficult. These discrepancies, however, were of sufficient magnitude to warrant a re-examination of the subject *ab initio* and this has been undertaken by authority of Congress in the Bureau of Chemistry of the Department of Agriculture. It is too early yet to announce the results of these investigations, although some of them have been completed, yet none of the data obtained have been thoroughly sifted and studied. There are certain principles, which become more firmly established as the work progresses and to which it would seem advisable to call the particular attention of the medical profession. These may be enumerated as follows:

First. Foreign substances of any kind, either for preserving, coloring or other purposes, should never be added to foods unless the exigency of such addition is paramount.

Second. The consumer and the experimenter should not be burdened with the proof of the harmlessness of such added bodies. The burden of proof in these cases justly falls on those who make the additions and the right to make them can only be established by demonstrating their permanent necessity and the harmlessness of the added ingredients.

Third. When the points mentioned in the above paragraph are not established the use of any bodies of this kind in food products should be plainly stated to the purchaser by appropriate labeling, which should be as prominent and characteristic as any other part of the label.

Fourth. Food products which contain these bodies without notification upon the labels should be forbidden or restricted in sale, both because of the deception practiced upon the purchaser by selling him substances for which he does not ask and because of the possible harm which may come to him in the consumption of such substances.

It appears to me that the above general principles pretty well cover the whole subject of the use of these bodies in foods. I might add, however, that the mere proof of harmlessness itself would not be sufficient justification for the use of preservatives and other added bodies. They must be shown to be positively beneficial and so clearly that it may appear without question that those foods which do not contain them are less nutritious, less desirable and less beneficial than those which do.

While I am not a prohibitionist in such matters, I feel strongly on this point, namely, that the public should not be deceived in any respect in regard to the character of the food products purchased.

In cases of sickness the medical profession is again interested in these matters because of foods which are prescribed for the invalid and the convalescent. These foods certainly should not contain substances of the nature of drugs, the presence of which is unknown to the physician prescribing them. For example, the case of ice-cream, which is often prescribed by physicians, may be cited. What physician, may I ask, will be able to tell the character of the food his patient may receive under a prescription for ice-cream? Instead of getting the pure nutritious cream and pure sugar with a harmless flavoring matter, the cream may be a varied assortment of coal-tar dyes, starch, gelatine, ptomaines, coloring-matter of all descriptions and the amount of real cream contained in the article may be nil or reduced to a very small quantity. This is only an extreme illustration, but shows the importance of knowing the purity of all food products, not only when used for general purposes of nutrition, but also when prescribed in cases of sickness by the attending physician.—*Central States Medical Magazine.*

